# Diet-induced changes in titer support a discrete response of *Wolbachia*-associated plastic recombination in *Drosophila melanogaster*

**DOI:** 10.1093/g3journal/jkab375

**Published:** 2021-11-15

**Authors:** Sabrina L Mostoufi, Nadia D Singh

**Affiliations:** Department of Biology, Institute of Ecology and Evolution, University of Oregon, Eugene, OR 97403-5289, USA

**Keywords:** *Drosophila melanogaster*, *Wolbachia pipientis*, plastic recombination, bacterial titer, host diet

## Abstract

Plastic recombination in *Drosophila melanogaster* has been associated with a variety of extrinsic and intrinsic factors such as temperature, starvation, and parasite infection. The bacterial endosymbiont *Wolbachia pipientis* has also been associated with plastic recombination in *D. melanogaster*. *Wolbachia* infection is pervasive in arthropods and this infection induces a variety of phenotypes in its hosts, the strength of which can depend on bacterial titer. Here, we test the hypothesis that the magnitude of *Wolbachia*-associated plastic recombination in *D. melanogaster* depends on titer. To manipulate titer, we raised *Wolbachia*-infected and uninfected flies on diets that have previously been shown to increase or decrease *Wolbachia* titer relative to controls. We measured recombination in treated and control individuals using a standard backcrossing scheme with two X-linked visible markers. Our results recapitulate previous findings that *Wolbachia* infection is associated with increased recombination rate across the *yellow-vermillion* interval of the X chromosome. Our data show no significant effect of diet or diet by *Wolbachia* interactions on recombination, suggesting that diet-induced changes in *Wolbachia* titer have no effect on the magnitude of plastic recombination. These findings represent one of the first steps toward investigating *Wolbachia*-associated plastic recombination and demonstrate that the phenotype is a discrete response rather than a continuous one.

## Introduction

Phenotypic plasticity is the phenomenon by which a single genotype may produce multiple phenotypes in response to variable environmental stimuli. Plasticity is pervasive in nature, affecting a range of phenotypes like morphology, development, behavior, and reproduction in bacteria, plants, and animals (Fusco and Minelli 2010; Forsman 2015; Fox *et al.* 2019). Meiotic recombination has also been shown to be phenotypically plastic, where the proportion of recombinant offspring increases in response to environmental stimuli. Plastic recombination has been observed in a number of taxa and in response to different stimuli: yeast experience elevated recombination rates under nutrient stress (Abdullah and Borts 2001), *Arabidopsis* displays recombination plasticity when exposed to extreme temperatures (Francis *et al.* 2007; Saini *et al.* 2017; Lloyd *et al.* 2018; Modliszewski *et al.* 2018), infection causes increased recombination in mosquitoes (Zilio *et al.* 2018) and plants (Chiriac *et al.* 2006; Andronic 2012), and social stress is associated with plastic recombination in male mice (Belyaev and Borodin 1982).

Plastic recombination also has a rich history of study in the fruit fly, *Drosophila melanogaster*. Temperature was the first condition associated with plastic recombination in *D. melanogaster*, a phenomenon which has been well-characterized over the last century (Plough 1917, 1921; Stern 1926; Hayman and Parsons 1962; Grell 1978; Kohl and Singh 2018). Several other factors have been identified which induce plastic recombination in *D. melanogaster*, including maternal age (Bridges 1927; Priest *et al.* 2007; Hunter *et al.* 2016a), starvation (Neel 1941), heat shock (Zhong and Priest 2011; Jackson *et al.* 2015), and parasite infection (Singh *et al.* 2015).

More recently, infection with the bacteria *Wolbachia pipientis* has been associated with plastic recombination in *D. melanogaster* (Singh 2019). *Wolbachia* is a gram-negative endosymbiont that infects approximately 40% of terrestrial arthropod species including insects, spiders, and mites (Zug and Hammerstein 2012). Though *Wolbachia* is found throughout the somatic and germline tissues of its hosts (for review see Pietri *et al.* 2016), it is particularly abundant in germ cells and is maternally inherited through the oocyte (Dobson *et al.* 1999; Clark et al. 2002). Different *Drosophila* species are infected with unique strains of *Wolbachia*, each with varied effects on host biology (for review see Serbus *et al.* 2008; Werren *et al.* 2008; Correa and Ballard 2016; Kaur *et al.* 2021). One of the most well-studied *Wolbachia*-associated phenotypes is cytoplasmic incompatibility, which causes certain mating pairings between infected and uninfected flies to produce nonviable embryos (Turelli and Hoffmann 1995). Other strains of *Wolbachia* can cause phenotypes like male offspring killing or decreased lifespan in *Drosophila* (Hurst *et al.* 2000; Chrostek and Teixeira 2015). The native *Wolbachia* strain in *D. melanogaster*, *w*Mel, has been shown to provide protection against viral pathogens (Hedges *et al.* 2008; Teixeira et al. 2008), increase host fecundity (Fry *et al.* 2004; Fast et al. 2011), and now is associated with plastic increases in recombination rate (Singh 2019; Bryant and Newton 2020).

Since *Wolbachia’*s role in plastic recombination is a recent discovery, there remains a large gap in our understanding of this interaction. One of the first papers to identify this phenomenon observed a correlation between *Wolbachia* infection and increased recombination across an interval of the X chromosome, but not on chromosome 3 (Hunter *et al.* 2016b). This finding was experimentally validated and expanded upon to demonstrate that *Wolbachia’*s effect on recombination was plastic and occurred in multiple strains of *D. melanogaster* (Singh 2019). Yet the scope, magnitude, and mechanisms behind this phenomenon are unclear.

Of particular interest is the potential effect of magnitude in *Wolbachia*-associated plastic recombination. Plastic phenotypes can often be described as either categorical, where the phenotype exists in discrete forms, or continuous, where the phenotype may display dose-dependency and scale with the magnitude of extrinsic or intrinsic factors (Scheiner and Levis 2021). Plastic recombination in *D. melanogaster* has displayed dose-dependency in response to temperature changes, where increased exposure time to heat shock continuously increased the magnitude of plastic recombination (Jackson *et al.* 2015). This raises an interesting question of how plastic recombination may be influenced by the strength of *Wolbachia* infection.

An obvious candidate for testing this question of magnitude is the number of bacteria present within a cell, referred to as titer. *Wolbachia*-associated phenotypes can vary according to bacterial titer, including cytoplasmic incompatibility (Calvitti *et al.* 2015), lifespan reduction (Chrostek and Teixeira 2015), and viral pathogen protection (Chrostek et al. 2013; Ye *et al.* 2016). These phenotypes are considered dose-dependent because the strength of the phenotype continuously scales with the number of *Wolbachia* cells present within the host. However, some *Wolbachia*-associated phenotypes may display both categorical and continuous responses; at low bacterial titer, the phenotype exists in discrete forms which are not expressed until a certain *Wolbachia* titer has been reached, after which the response scales continuously with increasing bacterial titer. This has been observed in both male-killing (Hurst *et al.* 2000) and lifespan reduction (Reynolds *et al.* 2003), where the phenotypes display both discrete and continuous responses.

If both plastic recombination and *Wolbachia*-associated phenotypes can display dose-dependency, this suggests that *Wolbachia-*associated plastic recombination may also follow the same pattern. Recently, Bryant and Newton (2020) tested this by using flies infected with two *Wolbachia* strains that maintain different titers and found that flies infected with a higher titer of *Wolbachia* also had a higher recombination rate. Though these results are consistent with the idea that *Wolbachia*-associated plastic recombination responds continuously to bacterial titer, it is difficult to determine since different *Wolbachia* strains were used. Because titer and *Wolbachia* strain were conflated, the distinct contributions of *Wolbachia* genotype and titer cannot be determined. Thus, additional research is needed to discern whether plastic recombination responds continuously to *Wolbachia* titer. It is also certainly possible that the response is continuous under some environmental conditions and discrete under others.

To address this question, we tested the effect of *Wolbachia* titer on plastic recombination in *D. melanogaster*. We used host diet to manipulate *Wolbachia* titer in fly ovaries under control, yeast-enriched, and sucrose-enriched conditions to evaluate the effect of titer on plastic recombination. Recombination rate was measured using classic genetic approaches in *Wolbachia-*infected and uninfected flies across a genomic interval on the X chromosome. Our data recapitulate that *Wolbachia* infection is associated with increased recombination rate and find that diet-induced changes in titer had no effect on the magnitude of plastic recombination. These findings demonstrate that *Wolbachia-*associated plastic recombination displays discrete phenotypes in response to diet-induced changes in *Wolbachia* titer in *D. melanogaster*.

## Materials and methods

### Fly strain and rearing

The *D. melanogaster* strain used in this experiment was RAL306, which comes from the *Drosophila* genetics reference panel (DGRP; Mackay *et al.* 2012; Huang *et al.* 2014). We used the RAL306 strain because it is naturally infected with *Wolbachia* and exhibits *Wolbachia*-associated plastic recombination (Hunter *et al.* 2016b; Singh 2019). To generate uninfected controls, we raised flies on tetracycline-containing media for two generations to remove *Wolbachia*. Tetracycline-containing media was created using standard cornmeal/molasses media mixed with ethanol-dissolved tetracycline at a final concentration of 0.25 mg/ml media (Holden *et al.* 1993). Following two generations of tetracycline treatment, flies were raised on standard media for over 10 generations to allow passive recolonization of the gut microbiome via the fly’s external microbiome.

We used PCR to confirm *Wolbachia* infection status prior to the start of the experiment. Briefly, single females were collected from stock vials of *Wolbachia*-infected and uninfected RAL306 flies. DNA was extracted with a standard squish protocol (Gloor and Engels 1992) and used in PCR with primers for the *Wolbachia* gene, *Wolbachia* surface protein (*wsp*), to identify the presence of *Wolbachia* (Jeyaprakash and Hoy 2000; Singh 2019; Supplementary Table S1).

### Diet treatments

For both *Wolbachia*-infected and uninfected groups, F1 virgin females were raised on one of three diet treatments: control, yeast-enriched, or sucrose-enriched. After 3 days, males were added to diet treatment vials with virgin females for crossing. We set up 10 replicate vials for each experimental group in a single block, repeated for four total blocks (Supplementary Figure S1).

To produce the sucrose-enriched diets, we made a 40% sucrose mixture following Serbus *et al.* (2015). Initially, we crossed flies in pure sucrose-enriched vials, but larvae raised on sucrose media showed increased mortality and slower development (unpublished observations). Therefore, we devised a strategy to allow adult flies to feed on the sucrose-enriched media while also promoting normal larval development by using “sucrose patties.” Sucrose-enriched mixture was poured into vials and allowed to cool before being sliced into 1 cm patties, which were placed on top of control diet vials. This strategy allowed adult flies to feed on the sucrose-enriched media while larvae could burrow down to feed on control media after hatching.

To make the yeast-enriched diets, we made a standard yeast paste by mixing dry active yeast and deionized water (Serbus *et al.* 2015). Approximately 2 ml of paste was added to control diet vials for the yeast-enriched treatments. Similar to the sucrose-enriched patties, this allowed adult flies to feed on yeast-enriched media while larvae could develop on control media.

### Experimental crosses

**Figure 1:**
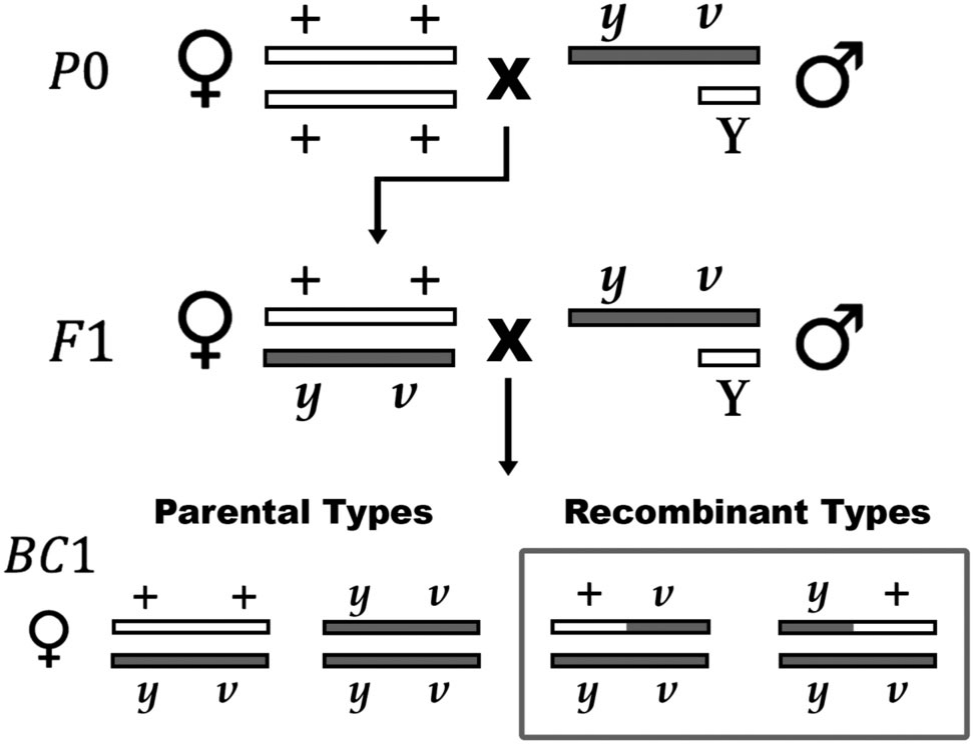
A two-step crossing scheme to measure recombination. Recombination rate can be estimated on the X chromosome using the recessive visible markers *yellow* (*y)* and *vermillion* (*v*) (33 cM). Males with the *y v* markers are crossed to wildtype (+ +) females. Heterozygous F1 females are backcrossed to the same male strain to produce BC1 progeny. Progeny which display either the yellow or vermillion phenotype are considered recombinant. Male BC1 genotypes are not shown, but males are heterogametic and require only one copy of the *yellow* or *vermillion* marker to display a phenotype.

Since *Wolbachia* have been shown to increase recombination on the X chromosome (Singh 2019), we measured recombination with a standard two-step backcrossing scheme using the markers *yellow* (*y*) and *vermillion* (*v*) (33 centimorgans (cM) apart) (Figure  1). In the first cross, roughly 20 RAL306 females and 20 *yv* males were crossed in 8 oz bottles. Heterozygous F1 virgin female offspring were collected from these bottles. For the second cross, 5 F1 females were backcrossed to 5 *yv* males in a vial, with approximately 10 vials per diet treatment per block, repeated for a total of 4 blocks (Supplementary Figure S1). BC1 offspring (Figure  1) were counted to estimate recombination rate in F1 females by calculating the recombinant fraction (cM/100), which is the proportion of recombinant types to the total number of offspring. For these crosses, recombinant types were heterozygous (female BC1) or hemizygous (male BC1) for either the *y* or *v* allele (Figure  1).

All crosses were conducted at 25°C with a 12:12 h light: dark cycle. Virgins were age-matched at approximately 48 h before crossing. In each cross, flies were allowed to mate and lay eggs for 4 days before being removed.

### Measuring *Wolbachia* titer

We collected and froze F1 females at −20°C after egg-laying for ovary dissections and DNA extraction. Flies from blocks 1–3 were dissected in 1x PBS for a total of 22 ovaries per experimental group. DNA was extracted from ovary samples using the DNeasy^®^ Blood & Tissue Kit (Qiagen) following insect and Gram-negative bacteria protocols. Quantitative PCR (qPCR) was conducted to amplify the *Wolbachia* gene, *wsp*, and estimate the average ovarian titer within each experimental group relative to two host genes, *αTub84B* and *CG15365* (Supplementary Table S1). Each experimental group consisted of seven technical replicates for *wsp* and four technical replicates for each of the host genes, along with nontemplate controls to assess qPCR reaction efficacy. We used the SYBR Green Mastermix and standard manufacturer’s protocols for qPCR on a QuantStudio3 Real-Time PCR System (Life Technologies). We also estimated primer efficiencies using a 1:5 serial dilution standard curve with five dilutions using DNA extracted from *Wolbachia*-infected flies.

### Statistical analyses

Recombination rate between groups was compared using a logistic regression model to evaluate statistical significance of the effect of *Wolbachia* infection ($W_{j}$), diet ($D_{i}$), or *Wolbachia* by diet interaction effects ($D_{i}\times W_{j}$). The full model is as follows, where *Y* refers to observed recombination data, μ refers to overall mean recombination rate, and ε refers to random variation:$\;Y_{i,j}=\mu +D_{i}+W_{j}+D_{i}\times W_{j}+\varepsilon$, (for i=1…3, j=1…2). We used the statistical software JMP Pro (v16.0.0) for logistic regression modeling, using a general linear model with binomial distribution and link logit function.

All other statistical analyses were carried out in RStudio (v1.2.5033). Mutant markers were tested for viability defects using *G*-tests for goodness of fit. A one-way ANOVA and Tukey’s multiple comparisons test were used to analyze differences in fly fecundity between experimental groups. A *post-hoc* analysis of recombination rate variance was conducted using a modified robust Brown-Forsythe Levene-type test and Tukey’s multiple comparisons test. For qPCR, raw Cq scores were analyzed using the Livak and Pfaffl methods (Livak and Schmittgen 2001; Pfaffl 2001) and differences between groups were tested using a one-way ANOVA and Tukey’s multiple comparisons test. The significance threshold for all statistical tests was set at 0.05.

Power analyses for recombination rate comparisons were conducted using the R package “SIMR” to validate experimental results (Green and Macleod 2016). Simulated data were generated in R to produce a range of differences in mean recombination rate between groups, which were tested using repeated simulations in SIMR to calculate statistical power, where 80% power or greater is considered ideal.

## Results

### Fly fecundity

**Figure 2:**
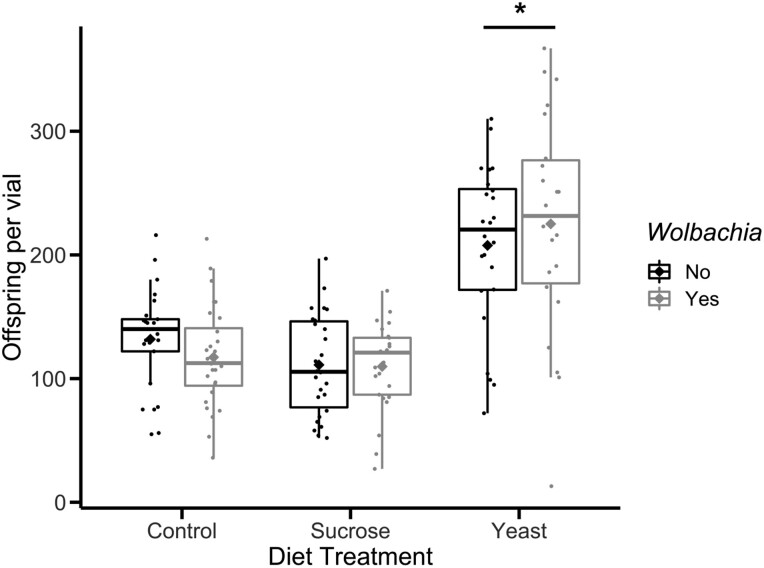
Fecundity, or number of offspring per vial, of experimental groups. *Wolbachia-*infected flies are shown in gray, while uninfected flies are shown in black. Each point corresponds to the total number of offspring in a single vial. Boxplots present summary statistics, where the top and bottom edges encompass the first to third quartiles and the middle bar represents the median for each group. Boxplot whiskers extend to the smallest and largest nonoutliers. The diamond in each boxplot represents the mean fecundity for each group. Statistically significant groups (*P < *0.05) are denoted with an asterisk (*).

To assess the effect of *Wolbachia* titer on plastic recombination, we set up crosses for *Wolbachia*-infected and uninfected flies on three diet treatments and measured recombination between the *yellow* and *vermillion* interval on the X chromosome. In total, 22,228 BC1 flies were scored for recombination (Table  1). For flies fed a control diet, the number of progeny per vial for *Wolbachia*-infected flies averaged 110 flies/vial, while uninfected flies averaged 111 flies/vial. On a sucrose-enriched diet, *Wolbachia*-infected flies produced an average of 117 flies/vial, compared to uninfected flies, which produced an average of 132 flies/vial. Finally, the number of progeny per vial for flies fed a yeast-enriched diet averaged 225 flies/vial, while uninfected flies averaged 208 flies/vial (Figure  2). Results from a one-way ANOVA test demonstrated that diet treatment [*P < *2e−16, ANOVA (*N* = 150, df = 2)], but not *Wolbachia* infection [*P = *0.942, ANOVA (*N* = 150, df = 1)] significantly affected fly fecundity. Further analysis with a Tukey’s multiple comparisons test found that the yeast-enriched diet increased fecundity significantly compared to the control (*P *<* *1.3e−13) and sucrose diet treatments (*P *<* *3.1e−14).

**Table 1:** Offspring counts for experimental groups

Diet treatment	*Wolbachia*-infected	Uninfected	Total
Control	3,284	3,296	6,580
Sucrose	2,824	2,888	5,712
Yeast	4,952	4,984	9,936
Total	11,060	11,168	22,228

### Viability effects of mutant markers

To determine whether the viability of the mutant markers affected the ratios of offspring phenotypes, we performed G-tests for goodness of fit within each vial for the following ratios: males versus females, wildtype (*wt*) flies versus *yv* flies, and *yellow* flies versus *vermillion* flies. The null hypothesis is a 1:1 ratio for all phenotypic classes compared. Significant deviations from expected ratios would indicate that the markers affected the viability of certain phenotype combinations, which would negatively impact recombination rate estimates.

Similar to previous work (Hunter *et al.* 2016b; Singh 2019), we find small but nonsignificant viability defects associated with these markers. Out of 151 crosses, seven showed significant deviation with regards to the male-female ratio, 11 deviated from expected wildtype to *yv* ratios, and nine deviated from the expected ratio of *yellow* to *vermillion* flies. However, after using the Bonferroni correction for multiple tests, only one of the deviant crosses remained significant (*P *=* *1.14 E-12, *G*-test). This specific cross had a ratio of 9.8 wildtype flies to *yv* flies and a recombinant fraction of 0.05. This likely stems from mating contamination and we discarded this cross from further analyses.

### Host diet and quantitative PCR

To compare *Wolbachia* titer between diet treatment groups, we ran qPCR with DNA extracted from frozen female F1 flies collected after egg-laying. Results are shown in Table  2, where gene expression of *wsp* relative to host genes in *Wolbachia*-infected flies was calculated using the Livak and Pfaffl methods (Livak and Schmittgen 2001; Pfaffl 2001). Analysis of qPCR data using either the Livak or Pfaffl method produced similar results, where flies fed a sucrose-enriched diet had the highest relative gene expression of *wsp* compared to control group flies and flies on the yeast-enriched diet (Table  2; Supplementary Figure S2). Since *wsp* expression is correlated with *Wolbachia* titer, this corresponds to a 3% increase in *Wolbachia* titer in flies on the sucrose diet treatment and a 23% decrease in *Wolbachia* titer in flies on the yeast diet treatment. Relative gene expression of *wsp* was significantly affected by diet treatment for both the Livak [*P* *= *0.0019, ANOVA (*N* = 21, df = 2)] and Pfaffl analysis methods [*P* *= *0.005, ANOVA (*N* = 21, df = 2)]. A Tukey’s multiple comparisons test indicated that *Wolbachia* titer was significantly reduced in the yeast-enriched diet treatment compared to flies in the control (*P* *= *0.008 Livak*, P* *= *0.018 Pfaffl) and sucrose-enriched diets (*P* *= *0.003 Livak*, P* *= *0.007 Pfaffl).

**Table 2:** Relative gene expression of WSP in fly ovaries

Diet treatment	Livak	Pfaffl
Control	1.091	1.012
Sucrose	1.122	1.044
Yeast	0.842*	0.799*

* *P *<* *0.05, Tukey’s multiple comparisons.

### The effect of infection and diet on recombination

**Figure 3:**
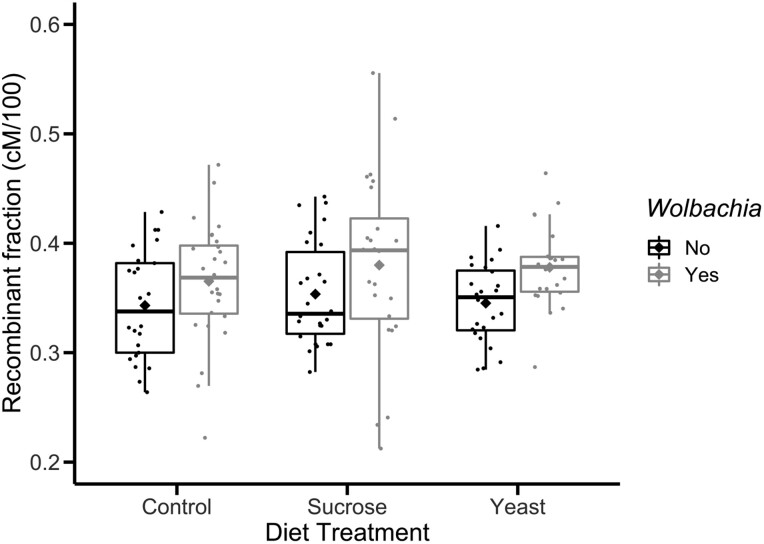
Recombination rate, reported as recombinant fraction, of experimental groups. The recombinant fraction is the proportion of recombinant progeny compared to the total number of progeny produced for each cross, which is equivalent to cM divided by 100. *Wolbachia-*infected flies are shown in gray, while uninfected flies are shown in black. Each point corresponds to the recombinant fraction of a single vial. Boxplots present summary statistics, where the top and bottom edges encompass the first to third quartiles and the middle bar represents the median for each group. Boxplot whiskers extend to the smallest and largest nonoutliers. The diamond in each boxplot represents the mean recombination rate for each group. Statistical significance was tested using a general linear model, where *Wolbachia* and experimental block significantly affected recombination rate (*P < *0.05), while diet and *Wolbachia*-diet interactions were not significant.

We used logistic regression modeling to identify variables which significantly contributed to differences in mean recombination rate between experimental groups. Results are shown in Table  3, where *Wolbachia* infection [*P* *= *0.0008, *X*^2^ test (*N* = 150, df = 1)] and experimental block [*P* *= *0.0001, *X*^2^ test (*N* = 150, df = 3)] were significantly associated with differences in recombination rate. We measured recombination rate across the *y—v* interval (33 cM) of the *X* chromosome, with an expected recombination rate of 30–35 cM. The effect of *Wolbachia* infection can be seen clearly in Figure  3, where *Wolbachia*-infected flies display an average recombination rate of 37.1 cM while uninfected flies display an average rate of 34.7 cM, resulting in an average increase of 2.4 cM in recombination rate across all diet treatments. Neither host diet [*P* *= *0.42, *X*^2^ test (*N* = 150, df = 2)] nor infection by diet interaction effects [*P* *= *0.43, *X*^2^ test (*N* = 150, df = 2)] was significant. Based on the power of our tests, we would have been able to detect a difference of 5.8% or greater between group means, which corresponds to a difference in recombination rate of approximately 2 cM (Supplementary Figure S3). This indicates that the effect of diet or *Wolbachia* titer, if present, was weaker than the effect of *Wolbachia* infection alone.

**Table 3:** Results of logistic regression modeling on recombinant fraction

Source	df	L-R χ^2^	Prob χ^2^
*Wolbachia*	1	11.315	0.0008*
Diet	2	1.723	0.42
*Wolbachia* *Diet	2	1.686	0.43
Block	3	20.435	0.0001*

* *P *<* *0.05, general linear model.

Since the sucrose diet treatment did not significantly increase *Wolbachia* titer relative to the control diet, we performed additional logistic regression modeling on the control and yeast diet treatment groups. *Wolbachia* infection [*P = *0.0001, *X*^2^ test (*N* = 99, df = 1)] and experimental block [*P = *0.038, *X*^2^ test (*N* = 99, df = 3)] were significantly associated with recombination rate differences, while host diet [*P = *0.26, *X*^2^ test (*N* = 99, df = 1)] and infection by diet interaction effects [*P = *0.97, *X*^2^ test (*N* = 99, df = 1)] were not significant (Supplementary Table S2).

We also tested for the effect of *Wolbachia* infection, titer, and diet on recombination rate variance, which was calculated as absolute residuals. Uninfected flies showed no significant difference in recombination rate variance between diet treatment groups [*P = *0.25, Levene’s test (*N* = 75, df = 2)], and a comparison between uninfected and *Wolbachia*-infected flies was also nonsignificant [*P = *0.11, Levene’s test (*N* = 150, df = 1)]. However, *Wolbachia*-infected flies displayed significant differences in variance between diet treatment groups [*P = *0.007, Levene’s test (*N* = 75, df = 2)] and a Tukey’s multiple comparisons test found that infected flies on a sucrose-enriched diet were significantly different from flies on a control (*P = *0.03) and yeast-enriched diet (*P = *0.003).

## Discussion

### Effect of *Wolbachia* infection and diet on recombination

The goal of this experiment was to assess whether *Wolbachia*-associated plastic recombination in *D. melanogaster* is continuous or discrete in response to changes in bacterial titer. *Wolbachia* cannot currently be transgenically modified, making it impossible to use genetic engineering to test for differences in titer. Several other factors have been shown to alter *Wolbachia* titer, including temperature (Hurst *et al.* 2000; Moghadam *et al.* 2018), bacterial genotype (Chrostek and Teixeira 2015), and host diet (Serbus *et al.* 2015). However, both temperature (*e.g.*, Plough 1917; Grell 1978; Jackson *et al.* 2015) and bacterial genotype (Singh *et al.* 2015; Bryant and Newton 2020) also affect recombination rate in *D. melanogaster*. Host diet can alter *Wolbachia* titer within fly ovaries, specifically that a yeast-enriched diet decreases titer while a sucrose-enriched diet increases titer (Serbus *et al.* 2015; Camacho *et al.* 2017; Christensen *et al.* 2019). Therefore, we used host diet to manipulate *Wolbachia* titer and tested the effects of *Wolbachia* infection, host diet, and *Wolbachia* titer on recombination rate. We find that *Wolbachia* infection is associated with a significant increase in recombination rate across the *y—v* interval on the X chromosome (Table  3). Our data indicate that the *Wolbachia*-associated increase in recombination is robust with regards to variation in host diet, as *Wolbachia*-infected flies displayed a higher recombination rate than their uninfected counterparts in each diet treatment (Figure  3). This finding adds to a growing body of literature which supports *Wolbachia* as an inducer of plastic recombination in *D. melanogaster* (Hunter *et al.* 2016b; Singh 2019; Bryant and Newton 2020).

Since we used host diet to manipulate *Wolbachia* titer, we also assessed whether diet treatments had an impact on plastic recombination. We find that our diet treatments did not significantly affect recombination rate in *Wolbachia*-uninfected flies (Table  3, Figure  3). The effect of diet on plastic recombination in flies has been severely understudied, where one previous study reported that starvation in larvae was associated with increased recombination rate (Neel 1941). Differences between our study and the previous one may indicate that only severe changes in diet such as starvation are sufficient to induce plastic recombination in *D. melanogaster*. However, it should also be noted that Neel’s study was carried out using markers on chromosome 3 (1941) while our study assessed recombination on the X chromosome. This may suggest that diet-associated plastic recombination is variable across the genome, as is the case for other conditions associated with plastic recombination such as temperature and *Wolbachia* infection (Grell 1978; Singh 2019). Outside of the present study, no recent investigations have been made into how starvation or diet affects recombination in flies, highlighting a need for additional research into the role diet may play in plastic recombination. Our study only uses three diet treatments, while a more rigorous investigation of the effects of varying levels of carbohydrates, proteins, and caloric content is needed to definitively assess the effect of diet on plastic recombination in flies.

### Effect of *Wolbachia* infection and diet on fecundity

Though our diet treatments did not affect recombination rate, there was an effect of diet on fecundity. We observed that the average number of offspring per vial was significantly different between diet treatments, with yeast-fed flies displaying the highest average fecundity (Figure  2). The influence of diet on lifespan and fecundity in *D. melanogaster* has been well-characterized, especially regarding sucrose and yeast content (Drummond-Barbosa and Spradling 2001; Bass *et al.* 2007). Specifically concerning fecundity, yeast-enriched diets greatly increase female fecundity, while sucrose-enriched diets decrease female fecundity (Bass *et al.* 2007).

*Wolbachia* are often associated with increased fecundity in host fly species (Weeks *et al.* 2007; Mazzetto *et al.* 2015; Singh 2019), yet we found no significant effect of *Wolbachia* infection on fecundity. However, this may reflect a strain-specific response, rather than the effect of *Wolbachia* infection on *D. melanogaster* as a whole. Differences in fly fecundity depend on *Wolbachia* genotype (Gruntenko *et al.* 2019), host genotype (Fry *et al.* 2004), and bacterial-host interactions (Singh 2019). For instance, the strain used in this experiment, RAL306, was also used in a study which reported an overall effect of *Wolbachia* infection on fecundity across multiple strains (Singh 2019). However, when examined individually, *Wolbachia*-infected RAL306 flies had a lower mean fecundity than uninfected RAL306 flies (Singh 2019). This suggests that *Wolbachia* broadly impacts fecundity, but this effect may vary with host genotype.

### Effect of *Wolbachia* titer on recombination rate

By using host diet to manipulate *Wolbachia* titer, we tested the effect of titer on the magnitude of *Wolbachia*-associated plastic recombination. We measured *wsp* gene expression relative to host genes to measure *Wolbachia* titer in infected fly ovaries for each diet treatment group. Our results agree with other studies which find that yeast-enriched diets decrease *Wolbachia* titer and sucrose-enriched diets increase *Wolbachia* titer in fly ovaries (Table  2; Serbus *et al.* 2015; Christensen *et al.* 2019). Our yeast diet treatment had a much stronger effect on *Wolbachia* titer than our sucrose diet treatment, resulting in a 23% decrease in titer compared to control flies, while flies on a sucrose diet showed a 3% increase in titer compared to control flies.

Combined with the recombination analysis which found no effect of infection by diet interactions (Table  3), these results suggest that changes in *Wolbachia* titer did not induce a continuous response in plastic recombination. This is, perhaps, not surprising in the case of the sucrose diet treatment, since a small increase in *Wolbachia* titer may not be enough to significantly affect the host fly’s biological processes. However, reanalysis of the data using only the control and yeast diet treatment groups still finds that *Wolbachia* infection significantly impacted recombination rate, but *Wolbachia* titer did not (Supplementary Table S2). So, while the yeast diet treatment significantly decreased *Wolbachia* titer, this decrease did not lower recombination rate relative to *Wolbachia*-infected control flies, yet still resulted in an increase in recombination rate relative to uninfected flies. These results provide us with several new pieces of information about *Wolbachia-*associated plastic recombination, which are discussed in more detail below.

Though *Wolbachia* titer did not affect the magnitude of recombination, it did influence recombination rate variance. *Wolbachia*-infected flies fed a sucrose-enriched diet, to promote high *Wolbachia* titer, had significantly greater variance than *Wolbachia*-infected flies on either a control or yeast-enriched diet. This finding suggests that increased *Wolbachia* titer may increase recombination rate variation, rather than increase the average rate of recombination beyond that caused by standard *Wolbachia* infection. Changes in variance have not previously been reported for other inducers of plastic recombination in *D. melanogaster*, nor for other *Wolbachia*-associated host phenotypes, suggesting that this may be a unique feature of *Wolbachia*-associated plastic recombination. This finding inspires multiple questions for future research, including why low *Wolbachia* titer did not result in decreased variance and whether this phenomenon is robust in response to other modifiers of *Wolbachia* titer.

### Discrete phenotypic responses

Based on our results, there are several new pieces of information we can conclude about *Wolbachia-*associated plastic recombination. First, the phenotype must require relatively large changes in bacterial titer to elicit a corresponding change in response. Neither the sucrose diet treatment group nor the yeast diet treatment group significantly affected recombination rate relative to controls. This suggests that changes in titer need to be more dramatic than what we observed (3%–23%) to potentially affect recombination rates. It is possible that these changes in *Wolbachia* titer caused small, nonsignificant changes in recombination rate; if so, these changes are smaller than the effect of *Wolbachia* infection alone. This suggests that this phenotype displays discrete rather than continuous responses, where large changes in *Wolbachia* titer are required to cause the magnitude of plastic recombination to increase. It is also interesting that the yeast diet treatment decreased *Wolbachia* titer, but not enough to eliminate the *Wolbachia*-associated plastic recombination phenotype. Logic would suggest that there must be some minimum threshold of bacteria below which plastic recombination would not be induced in flies, but we did not reach that minimum in this experiment. Future work exploring even lower ranges in *Wolbachia* titer may be able to locate this threshold level.

If *Wolbachia*-associated plastic recombination displays discrete phenotypic responses, this follows the same trend as male-killing, another *Wolbachia*-driven trait in *Drosophila.* In *D. bifasciata*, *Wolbachia* infection causes increased mortality of male offspring, leading to modified sex ratios (Hurst *et al.* 2000). However, *Wolbachia* titer decreases in flies exposed to elevated temperatures, which causes male mortality rates to decrease and offspring sex ratios to return to normal (Hurst *et al.* 2000). These findings suggested that this phenotype requires a threshold level of *Wolbachia* to be expressed and displays discrete responses at low titers and continuous responses at high titers. The same may be true for *Wolbachia*-associated plastic recombination, where recombination is modified in discrete amounts in infected flies. It may also be true that *Wolbachia*-associated plastic recombination is continuous and dose-dependent, but only at titer levels more extreme than could be achieved through manipulations in host diet.

Another study looked at the effect of bacterial titer on plastic recombination using different strains of *Wolbachia* (Bryant and Newton 2020). They find that *D. melanogaster* infected with the *Wolbachia* strain *w*MelPop display a higher recombination rate across the *yellow-vermillion* interval of the X chromosome when compared to flies infected with a different *Wolbachia* strain, *w*Mel (Bryant and Newton 2020). The *w*MelPop strain maintains a much higher titer in flies, which could suggest that the magnitude of recombination corresponded with *Wolbachia* titer and indicates a dose-dependent relationship. Yet, as noted above, this study cannot separate the effect of titer from *Wolbachia* strain since two different strains were used in the experiment. Though *w*Mel is the native *Wolbachia* strain in *D. melanogaster*, *w*MelPop is considered pathogenic because it maintains a high titer and significantly decreases host lifespan (Strunov *et al.* 2013; Chrostek and Teixeira 2015). Other pathogenic bacteria have been shown to plastically increase recombination rate in *D. melanogaster* (Singh *et al.* 2015), making it difficult to say whether an increase in recombination rate in *w*MelPop-infected flies is due to bacterial titer, its pathogenic nature, additional genetic differences between the two bacterial strains, or a combination of factors.

Our data are consistent with the plastic recombinational response to *Wolbachia* infection being discrete, with even low bacterial titers inducing the response. It is certainly possible that larger changes in *Wolbachia* titer can induce different magnitudes of plastic recombination in the host. Future experiments which test a large range of *Wolbachia* titers are necessary to fully understand the nature of titer effects on *Wolbachia*-associated plastic recombination.

### The *Drosophila* microbiome

It may also be true that *Wolbachia*-associated plastic recombination is continuous and dose-dependent, but that this effect is masked in our study due to complex interactions between diet, host, and the microbiome. Diet is known to have a significant impact on microbiome composition in several species (Turnbaugh *et al.* 2008; Read and Holmes 2017; Erkosar *et al.* 2018). In *D. melanogaster*, diets rich in either yeast or sucrose caused significant changes in abundance of certain members of the gut microbiome (Chandler *et al.* 2011). These changes in microbiome composition can have drastic impacts on host biology including hormone production, metabolism, and nutrient acquisition (Leulier *et al.* 2017). Specific members of the *D. melanogaster* microbiome have been shown to support larval feeding under starvation conditions (Consuegra *et al.* 2020), suggesting that diet-induced changes in the microbiome can significantly impact host development. Though our results suggest that diet had no significant effect on recombination rate, as uninfected flies showed similar mean recombination rate for each diet treatment (Figure  3), it is difficult to rule out without directly measuring changes in microbiome composition.

In addition to the gut microbiome, which comes in direct contact with nutritional elements, host diet also affects *Wolbachia*. One finding our study takes advantage of is that increased sucrose or yeast in *D. melanogaster* diets can manipulate *Wolbachia* titer (Serbus *et al.* 2015). *Wolbachia* rely on their host to acquire nutrients, so changes in diet can affect microbe behavior and replication and may ultimately impact host biology. Yeast diets have been shown to affect *Wolbachia* cell physiology, which could influence the growth and behavior of the bacteria (Serbus *et al.* 2015). However, *Wolbachia* in flies fed the yeast diet treatment still produced the same increase in recombination rate as control flies, suggesting that changes in cell physiology did not impact plastic recombination. In addition, *Wolbachia*-infected *D. melanogaster* have been shown to alter behavior and diet preference, potentially as a strategy to reduce negative effects on lifespan and fecundity (Ponton *et al.* 2015; Truitt *et al*. 2018). Though flies may alter their behavior under these conditions, *Wolbachia* have been shown to have no effect on emergence time or host nutrition under starvation conditions (Harcombe and Hoffmann 2004). We saw no significant differences in fecundity or viability related to infection status in this study, but it is unclear whether *Wolbachia*-infected flies fed experimental diets experienced changes in behavior that may have impacted recombination estimates.

Finally, the gut microbiome and *Wolbachia* have been shown to influence one another. *Wolbachia* infection can alter relative abundances of members of the gut microbiome compared to uninfected flies (Simhadri *et al.* 2017). Conversely, ingestion of certain species of gut bacteria has been shown to influence *Wolbachia* abundance (Rudman *et al.* 2019). Taken together, these findings present a complex web of interactions between host, diet, the gut microbiome, and *Wolbachia*. Though it is difficult to estimate the impact of these interactions on our results, it remains clear that *Wolbachia*-associated plastic recombination is robust in response to both measured changes in diet and unmeasured changes in microbiome composition. Future work may focus on studying *Wolbachia*-only experimental flies, where germ-free flies are reinfected with *Wolbachia*, to remove potentially confounding variables caused by these complex interactions. However, there is also value in studying these systems in their natural state in order to gain a more complete understanding of native host-microbe associations.

## Conclusions

Our current inability to transgenically modify *Wolbachia* makes it impossible to assess the effect of titer alone on *Wolbachia*-associated phenotypes. Though differences in titer can be assessed through manipulation of host diet (Serbus *et al.* 2015), temperature (Hurst *et al.* 2000; Moghadam *et al.* 2018), or *Wolbachia* strain (Chrostek and Teixeira 2015), these methods include confounding variables which make it difficult to definitively assign *Wolbachia* titer as the causative agent in phenotypes of interest. Our present study controls for host and microbe genotype and finds that *Wolbachia*-associated plastic recombination is a phenotype with discrete responses, while acknowledging the ways in which changes in host diet may influence that finding. Future advances toward making genetic manipulation possible in *Wolbachia* would allow the role of titer to be more definitively tested without confounding effects.

## Data availability

Fly strains are available upon request. All raw data are available on Dryad: https://doi.org/10.5061/dryad.866t1g1qj.

Supplementary material is available at *G3* online.

## Supplementary Material

jkab375_Supplementary_Data
