# Supplementary material for: Diet-induced changes in titer support a discrete response of Wolbachia-associated plastic recombination in Drosophila melanogaster
Source: G3 (Bethesda). 2021 Nov 15;12(1):jkab375. doi: 10.1093/g3journal/jkab375 (PMC8728003; doi:10.1093/g3journal/jkab375)
Supplement: jkab375_Supplementary_Data [file jkab375_supplementary_data.docx]

**SUPPLEMENTARY MATERIALS**

| **Table S1: Primer information** | | | |  |
| --- | --- | --- | --- | --- |
| **Gene name** | **Product length** | **Sense strand** | **Antisense strand** | |
| *wsp* | 160 bp | 5' CATTGGTGTTGGTGTTGGTG 3' | 5' ACCGAAATAACGAGCTCCAG 3' | |
| *αTub84B* | 123 bp | 5' TCCTCGATTACCGCCTCTCTG 3' | 5' GATAACGGGGGCGTAGGTC 3' | |
| *CG15365* | 117 bp | 5' CGGGAGTCACAGCAACTTG 3' | 5' CTCAGCTCGAACATTTGTCGTAT 3' | |

| **Table S2: Results of logistic regression modeling on recombinant fraction for control and yeast diet groups** | | | |
| --- | --- | --- | --- |
| **Source** | **DF** | **L-R** $\boldsymbol{X}^{\boldsymbol{2}}$ | **Prob** $\boldsymbol{X}^{\boldsymbol{2}}$ |
| *Wolbachia* | 1 | 14.650 | 0.0001* |
| Diet | 1 | 1.267 | 0.2603 |
| *Wolbachia**Diet | 1 | 0.002 | 0.9685 |
| Block | 3 | 8.438 | 0.0378* |
| * = *P* < 0.05, general linear model | | | |

**
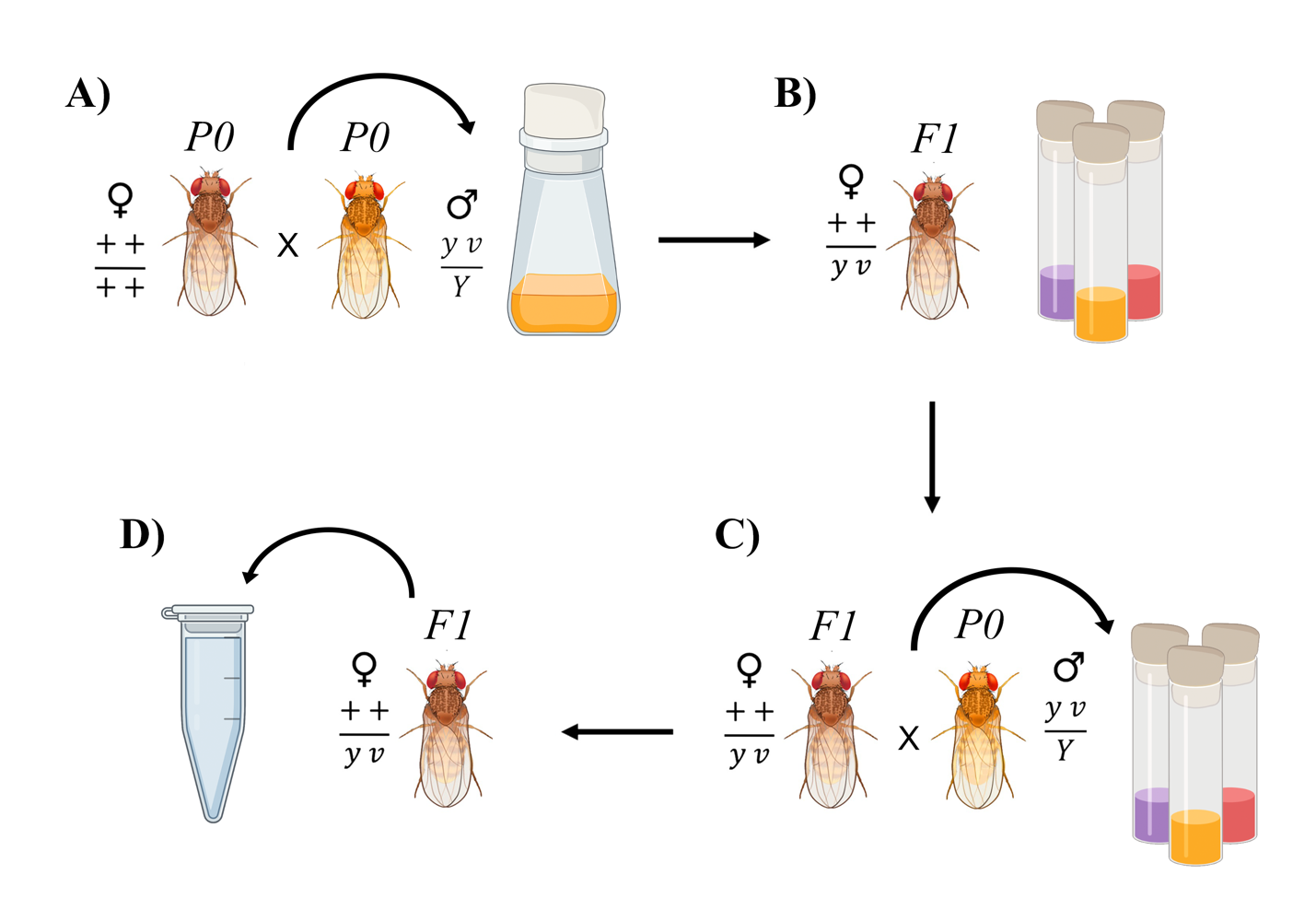
**

**Figure S1: Experimental design for diet treatments.** A) In the first generation (P0), 10 virgin RAL306 females and 10 *yellow-vermillion* (*yv*) males were crossed in 8oz bottles and allowed to lay eggs for 4 days. B) Virgin F1 females were collected from bottles and age-matched for 48 hours prior to diet treatment. For each diet treatment group (control, sucrose-enriched, and yeast-enriched), 10 vials were set up with 5 virgin F1 females per vial. Flies were kept on diet treatments for 3 days prior to crossing. C) 5 *yv* males were added to each vial for crossing with F1 virgin females. Flies were allowed to lay eggs for 4 days prior to removal. D) After egg laying was finished, F1 females were collected and stored in tubes at -20ºC until ready for DNA extraction and qPCR. Steps A-D constitute one experimental block, which was repeated for a total of 4 blocks for the entire experiment.

**
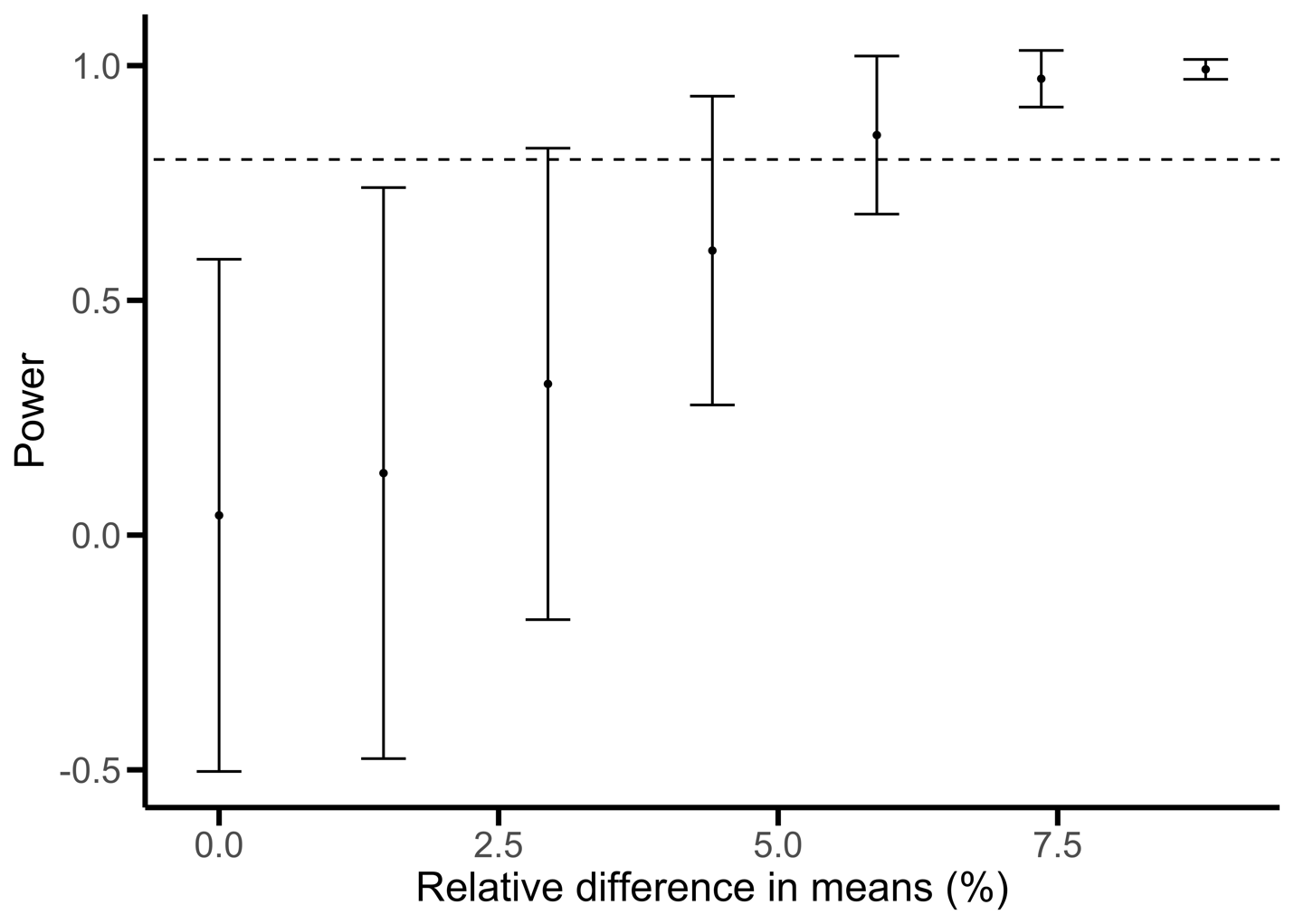
**

**Figure S2: qPCR results from *Wolbachia*-infected flies in experimental groups.** Results are presented as relative gene expression which was calculated using both the Livak and Pfaffl methods for qPCR analysis. Gene expression of *wsp* is relative to host genes, *aTub84B* and *CG15365*, and expression is then compared between experimental diet groups and the control diet group. Each point corresponds to the relative gene expression of a single sample. Boxplots present summary statistics, where the top and bottom edges encompass the first to third quartiles and the middle bar represents the median for each group. ﻿Boxplot whiskers extend to the smallest and largest nonoutliers. The diamond in each boxplot represents the mean gene expression for each group. Statistically significant groups (*P* < 0.05) are denoted with an asterisk (*).


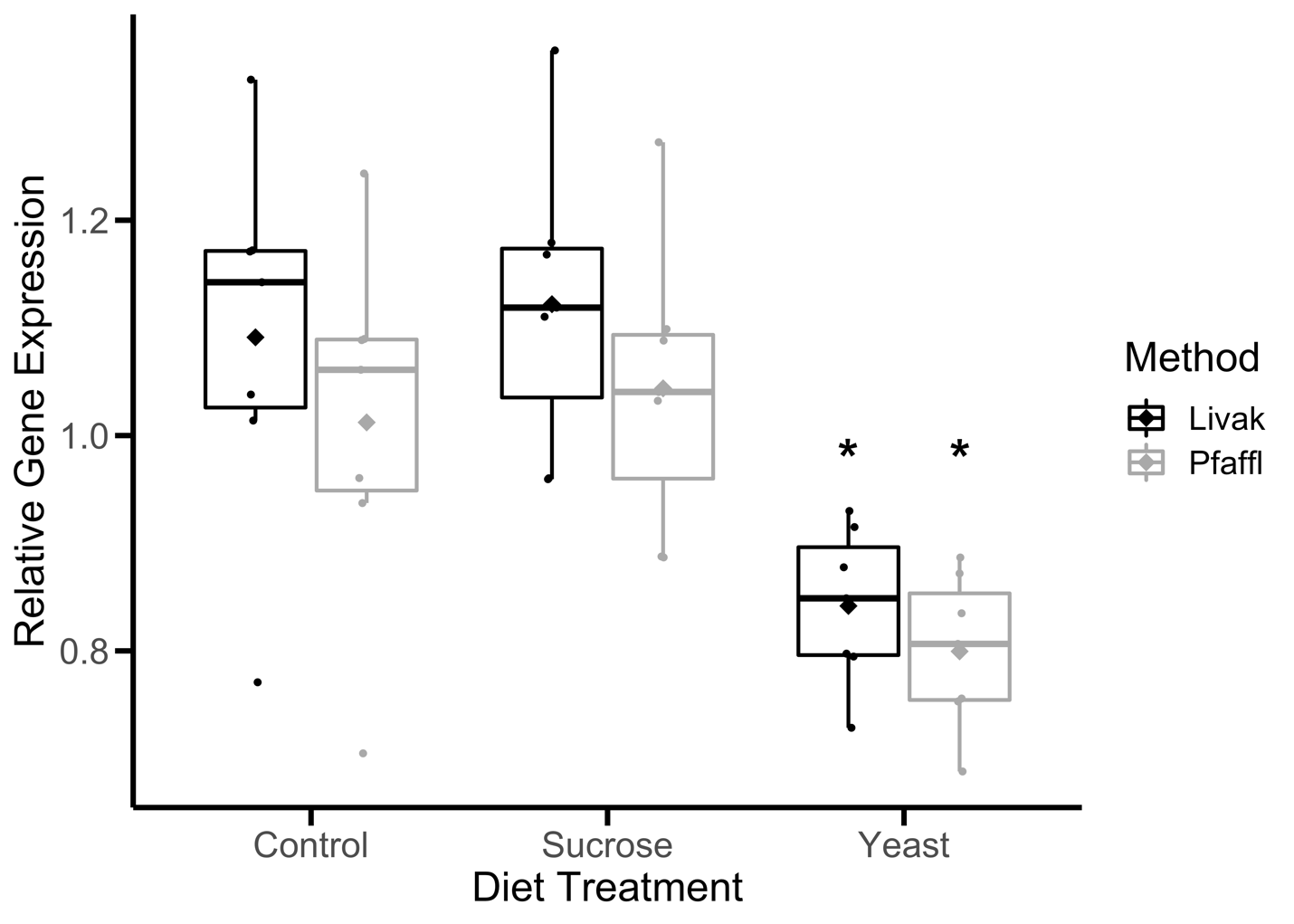


**Figure S3: Power analysis to validate recombination results.** Experimental data were simulated in R to create a range of differences in means between experimental groups. These data were then tested using the R package “SIMR”, which performed and compiled results from multiple rounds of statistical tests. Results are presented above, where each point corresponds to the power to detect a significant result in simulated data for each effect size and bars represent 95% confidence intervals. The dashed line is set at 0.80, which is the standard significance threshold for power analyses.
